# Genetic source tracking of an anthrax outbreak in Shaanxi province, China

**DOI:** 10.1186/s40249-016-0218-6

**Published:** 2017-01-17

**Authors:** Dong-Li Liu, Jian-Chun Wei, Qiu-Lan Chen, Xue-Jun Guo, En-Min Zhang, Li He, Xu-Dong Liang, Guo-Zhu Ma, Ti-Cao Zhou, Wen-Wu Yin, Wei Liu, Kai Liu, Yi Shi, Jian-Jun Ji, Hui-Juan Zhang, Lin Ma, Fa-Xin Zhang, Zhi-Kai Zhang, Hang Zhou, Hong-Jie Yu, Biao Kan, Jian-Guo Xu, Feng Liu, Wei Li

**Affiliations:** 1 Shaanxi Provincial Center for Disease Control and Prevention, Shaanxi province, China; 2 National Institute for Communicable Disease Control and Prevention, China CDC, Changping, Beijing China; 3 State Key Laboratory for Infectious Disease Prevention and Control, Beijing, China; 4 Collaborative Innovation Center for Diagnosis and Treatment of Infectious Disease, Hangzhou, China; 5 Division of Infectious Disease, China CDC, Beijing, China; 6 Institute of Military Veterinary, AMMS, Key Laboratory of Jilin Province for Zoonosis Prevention and Control, Changchun, Jilin China; 7 Yan’an Prefecture Center for Disease Control and Prevention, Yan’an, Shaanxi Province China

**Keywords:** Anthrax, Outbreak, *Bacillus anthracis*, Molecular typing, canSNP, MLVA, SNR, Shaanxi province, China

## Abstract

**Background:**

Anthrax is an acute zoonotic infectious disease caused by the bacterium known as *Bacillus anthracis.* From 26 July to 8 August 2015, an outbreak with 20 suspected cutaneous anthrax cases was reported in Ganquan County, Shaanxi province in China. The genetic source tracking analysis of the anthrax outbreak was performed by molecular epidemiological methods in this study.

**Methods:**

Three molecular typing methods, namely canonical single nucleotide polymorphisms (canSNP), multiple-locus variable-number tandem repeat analysis (MLVA), and single nucleotide repeat (SNR) analysis, were used to investigate the possible source of transmission and identify the genetic relationship among the strains isolated from human cases and diseased animals during the outbreak.

**Results:**

Five strains isolated from diseased mules were clustered together with patients’ isolates using canSNP typing and MLVA. The causative *B. anthracis* lineages in this outbreak belonged to the A.Br.001/002 canSNP subgroup and the MLVA15-31 genotype (the 31 genotype in MLVA15 scheme). Because nine isolates from another four provinces in China were clustered together with outbreak-related strains by the canSNP (A.Br.001/002 subgroup) and MLVA15 method (MLVA15-31 genotype), still another SNR analysis (CL10, CL12, CL33, and CL35) was used to source track the outbreak, and the results suggesting that these patients in the anthrax outbreak were probably infected by the same pathogen clone.

**Conclusions:**

It was deduced that the anthrax outbreak occurred in Shaanxi province, China in 2015 was a local occurrence.

**Electronic supplementary material:**

The online version of this article (doi:10.1186/s40249-016-0218-6) contains supplementary material, which is available to authorized users.

## Multilingual abstracts

Please see Additional file 1 for translations of the abstract into the five official working languages of the United Nations.

## Background

Anthrax is an acute infectious zoonotic disease caused by the spore-forming, aerobic, gram-positive, non-motile bacterium *Bacillus anthracis* (*B. anthracis*). *B. anthracis* can form spores resistant to extreme environmental conditions and can persist for long periods of time in soil or hay. Susceptible herbivores, such as cows, mules, sheep, horses, and donkeys, can become infected through contact with *B. anthracis* spores. Humans can become infected incidentally through contact with diseased animals or by inhaling spores from contaminated animal products [1]. There are three primary forms of the disease in humans, namely inhalation, gastrointestinal, and cutaneous anthrax. The most common form, cutaneous anthrax, accounts for 95% of cases worldwide [1]. It is mainly caused by the handling of infected animal carcasses or products of diseased animals. A new infection form, injection anthrax, had recently been discovered [2].

Efficient genotyping methods are required not only to assist microbial forensics to track outbreaks back to their origins, but also for facilitating epidemiological studies in order to reduce the risk of an outbreak or control it effectively if it does occur [3]. *B. anthracis* is a relatively homogeneous bacteria species with a demonstrated lack of polymorphism, which may be due to the long periods its endospores spend being dormant during its lifecycle [4]. Genetic markers such as single-nucleotide polymorphisms (SNPs) have been used to illustrate the phylogenetic and evolutionary relationship of *B. anthracis* strains, which allows us to better understand how they fit into regional and global phylogeographic patterns [4, 5]. An approach termed canonical single nucleotide polymorphisms (canSNP), which includes 13 selected SNPs located at the key phylogenetic junctions in the *B. anthracis* SNP tree, could replace the genome-wide SNP analysis [4]. Other molecular typing methods, such as the multiple-locus variable-number tandem repeat analysis (MLVA) [6–8], have also been used to illustrate the phylogenetic relationship of *B. anthracis* strains and for source tracking in the event of a bioterrorist attack or anthrax outbreak [3]. The MLVA15 scheme described by Van Ert et al. in 2007 [4] includes eight markers that were initially described by Keim et al. [9] and seven markers newly included in the study of Van Ert et al. [4]. The global genetic population structure of *B. anthracis* has previously been defined using canSNP typing and MLVA. It was found that the A.Br.001/002 subgroup was a major part of the *B. anthracis* population in China [4, 10]. Single-nucleotide repeats (SNRs), also called mononucleotide nucleotide repeats, are a special type of variable-number tandem repeats (VNTRs) that display very high mutation rates [11, 12]. A previous study showed that seven strains of *B. anthracis* recovered from Northeast China from 2010–2014 were distinguished as A.Br.001/002 (*n* = 5) or A.Br.Ames (*n* = 2) sublineages, and the two A.Br.Ames strains were further identified as same SNR pattern [13]. Because SNR markers can provide additional genetic resolution among *B. anthracis* strains of the same MLVA genotype [14, 15], the SNR analysis has therefore been used as a means of source tracking anthrax outbreaks [14].

Anthrax is an endemic disease and cases have been reported every year in China, especially in the northern and western provinces of mainland China. A strengthened national surveillance system covering seven provinces (expanded to 11 provinces in 2014) in mainland China has been in place since 2005. On 8 August 2015, an anthrax outbreak with 20 cutaneous anthrax cases was reported in Shaanxi province, which resulted in more than 300 people contacted with the contaminated animal products were under medical observation, and, at least 15 mules and two donkeys died. In order to determine the genetic relationship among the human cases and investigate the possible routes of transmission, the canSNP and the MLVA15 typing methods were employed, and their profiles were compared with the *B. anthracis* strains in the Chinese *B. anthracis* Genetic Information Database (CBaGID, Chinese Center for Disease Control and Prevention, CDC). In addition, a more precise method, the SNR analysis, was used to source track the outbreak.

## Methods

### Epidemiological investigation

In early August 2015, a suspected anthrax outbreak was reported to China’s National Health and Family Planning Commission. A national field team including clinical, epidemiologic, and laboratory personnel carried out an investigation and attempted to control the disease together with provincial and local CDC staff. A total of 20 clinical samples, including serum and blister fluid, were collected from each suspected individual during the investigation. A suspected cutaneous anthrax case in humans was defined as an acute onset of skin vesicles or skin ulceration with a raised margin and central black eschar and having corresponding exposure history (contacting infected animal product, contaminated fomite, etc.). Confirmed cases were determined through supportive laboratory tests, including isolation of bacilli, a four-fold increase in anti-protective antigen (PA) antibody titer. We also categorized suspected cutaneous anthrax cases as confirmed cases if they were evidenced by at least two of the following evidence: Morphology under the microscope with traditional Gram’s and M’Fadyean’s (polychrome methylene blue) stained smears in fresh tissue or blood samples, positive serology tests (ELISA or colloidal gold test), and positive real-time polymerase chain reaction (RT-PCR). This met the diagnostic criteria recommended by the World Health Organization (WHO) [16].

All procedures performed in studies were in accordance with the ethical standards of the institutional research committee in China CDC. The clinical samplings were obtained with the consent of related patients. The blood and meat from diseased and dead animals were sampled by local Animal Husbandry and Veterinary Inspection organization after permission was obtained from animal owners.

### Laboratory diagnosis

Two types of clinical specimens (blood and blister fluid) were collected from patients in hospital. Blister fluid was streaked on nutrient LB agar plates or blood agar plates and cultured at 28 °C for 24 h. Then, specimens were stained on slides with Gram and M’Fadyean staining. Finally, vesicular fluid samples of suspected cases were used to extract DNA templates according to the DNeasy Blood & Tissue Kit (QIAGEN Germany) manual. A RT-PCR reaction system (*B.anthracis* Test Kit〈real-time PCR〉, Shanghai Huirui Biotechnology Co., Ltd Shanghai), targeting the protective antigen gene (*pag*A), capsule synthesis gene (*cap*C), and chromosomal *rpoB* genes, was used in the laboratory diagnosis. The bacteria isolated from patients and animals were identified by culture, staining and microscopy examination, bacteriophage test*,* Colloid Gold Dip Stick test of *B. anthracis*, and Real-time PCR methods. The colloid gold method was also used to test antibody responses of the capsular antigen of *B. anthracis*. A four-fold increase against the PA antigen or a positive conversion by ELISA confirmed infection.

### DNA preparation

A *B. anthracis* strain was streaked onto LB agar plates and then incubated at 37 °C for 16–18 h. The single colonies were suspended in 0.5 ml of TE buffer (10.0 mM Tris–HCl [pH 8], 1. 0 mM EDTA) in 1.5 ml centrifugal tubes. Then, 200 μl of lysis buffer with 20 μl proteinase K mixture was added to the sample, which was incubated at 55 °C for 30 min to denature the protein and then incubated at 100 °C for a further 10 min. DNA from clinical specimens was extracted using the QIAamp® DNA Mini Kit (QIAGEN Germany), as per the manufacturer’s instructions. Chromosomal DNA was collected and filtered using 0.22 μm filter unites (Millipore, Bedford, MA, USA). Chromosomal DNA was diluted with sterile nuclease-free H_2_O (1:10) and used as a template for PCR amplification.

### canSNP and MLVA subtyping

The canonical SNP analysis with 13 markers was performed as described by Van Ert et al. [4, 5]. The MLVA15 analysis was performed as described by Van Ert et al. [4], with the following modifications on capillary electrophoresis (CE). The forward primers were labeled with different fluorescent dyes, FAM or HEX. The amplification was diluted with water to a ratio of 1:80 (for the amplification of specimens DNA extracted from vesicular fluid, not diluted for CE). The size of the amplicons was resolved by CE using an ABI PRISM® 3730xl genetic analyser (Applied Biosystems USA). The nomenclature of genotypes in the MLVA15 analysis was designated according to previously literatures [4]. For new genotypes of MLVA15, the nomenclatures labelled “CN” were employed.

### SNR analysis

We applied the SNR method as described by Kenefic et al. [12] with little modification, i.e., PCRs were performed in four singleplex reactions in a final volume of 25 μl with *Pfu* DNA polymerases, followed by CE using an ABI PRISM® 3730xl genetic analyser. Four primers (CL10, CL12, CL33, and CL35) described by Stratilo et al. [11] were used as the sequencing primers to identify the SNR locus sequences.

### Cluster analysis

Data from the canSNP and MLVA analyses were processed together using the BioNumerics software package (version 5.10, Applied Maths Belgium); these data merged into the CBaGID. Information regarding 15 VNTR profiles described in previous studies [4, 10, 13] was also merged into the database. There was information on a total of 420 isolates in the CBaGID, including our strains (*n* = 190) and isolates from previous research (*n* = 230). A total of 211 isolates, which belonged to the A.Br.001/002 and A.Br.Ames subgroups, were used in this study, out of which 106 strains were collected by the national anthrax surveillance system in China from 1953–2015. The unweighted pair-group method with arithmetic means and a categorical coefficient were used for the cluster analysis. Cluster analysis of the categorical data was presented as a dendrogram. Data from the SNR analysis was processed by the BioNumerics software and illustrated by minimum spanning tree (creation of complexes parameter: maximum and minimum neighbour distances were 2 and 1, respectively).

## Results

### Epidemiological investigation

**Fig. 1:**
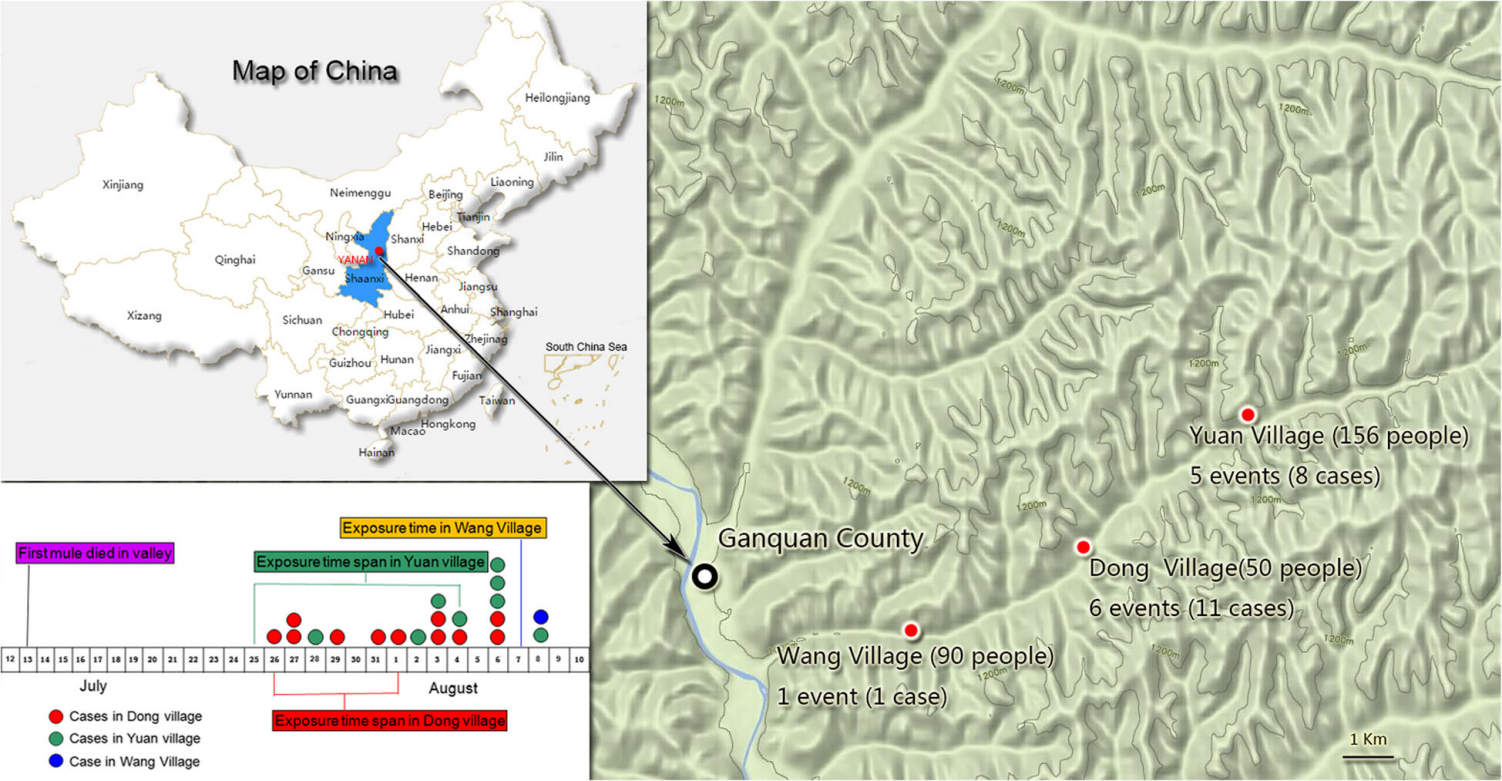
Exposure time and geographical distribution of case patients in Ganquan county, Shaanxi province, China

A total of 20 suspected cutaneous anthrax cases (four female and 16 male) were reported by the health authorities in Ganquan county, Yan’an prefectures, Shaanxi province, between 26 July and 8 August 2015. These cases were exposed to the anthrax in three villages located in the same mountainous valley (see Fig. 1). Seventeen cases butchered sick mules or handled the skin of a slaughtered animal, and three cases had contact with the carcass or meat of a sick animal. A total of 12 infectious events (co-exposure in skinning or slaughtering same sick animal) occurred in the valley. The suspected cutaneous anthrax cases had skin lesions characteristic of the disease. No case of suspected gastrointestinal anthrax was identified in nearby village farms when the search area was expanded. The index patient was a 66-year-old male farmer, who slaughtered a dead mule on 26 July, with cutaneous anthrax appearing on his fingers on the same day. One isolate was isolated from his vesicular fluid. However, no *B. anthracis* strain was isolated from the other patients, which may have been because the specimens were collected after antibiotics had already been administered in these patients for at least two days.

According to the criteria recommended by the WHO, a total of 19 confirmed cases were experimentally identified (one case with isolation of *B. anthracis* and a four-fold increase in the antibody titer; 15 cases with four-fold increase or positive conversions in the antibody titer and positive RT-PCR results; and three cases confirmed by two laboratory methods, i.e. the detectable anti-PA antibody with above 1:50 titer but no four-fold increase in the antibody titer and the positive RT-PCR result. One suspected case was not considered as confirmed case because clinical manifestations and laboratory evidence were lacking (see Additional file 2: Table S3). The overall attack rate was 6.7% (20/298).

Veterinarians in the investigation team inspected the sick and dead animals and gathered epidemiologic information by interviewing the owners of the animals. As early as 13 July 2015, a mule died after becoming ill, exhibiting bleeding of unclotted blood from nostrils and a bloated carcass. There were a total of 32 mules and donkeys in the outbreak area and livestock was not vaccinated routinely. No livestock were previously imported into the suffered valley by trading within one month before the outbreak occurred. A total of 15 mules and two donkeys died as a result of the outbreak, and at least 12 dead animals were butchered and skinned by villagers. Another 15 diseased animals (12 mules and three donkeys) in the valley were slaughtered and buried by 19 August after inspection by veterinarians. A total of five isolates of *B. anthracis* were isolated from the diseased and dead animals.

### The phylogenetic characteristics of outbreak-related *B. anthracis* strains in the CBaGID

**Fig. 2:**
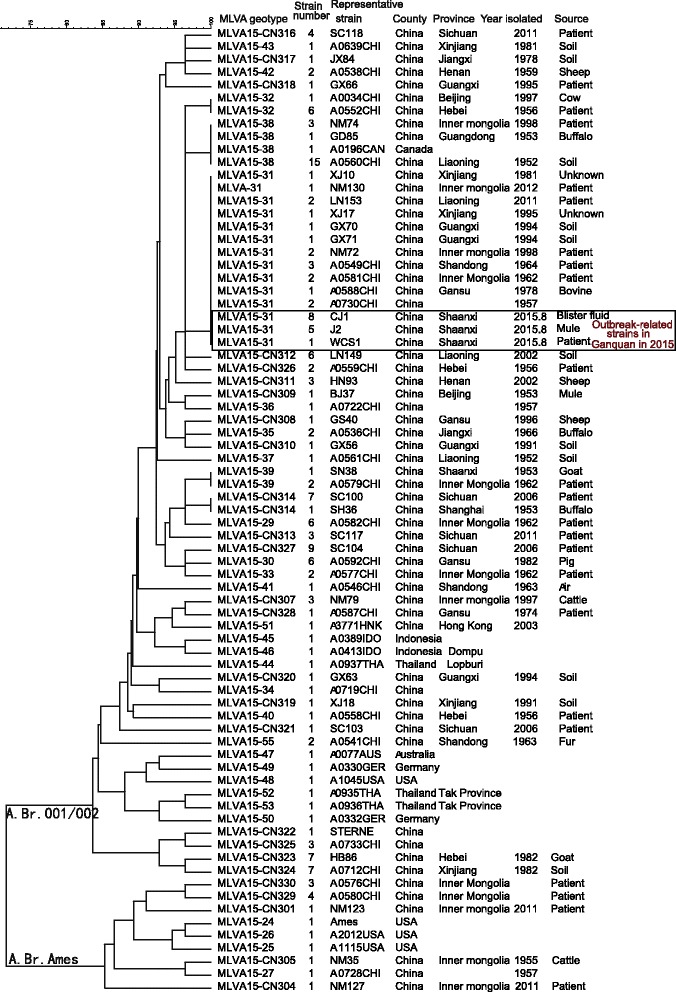
Dendrogram of canSNP typing and MLVA15 among outbreak-related strains in 2015, and representative stains of A.Br.001/002 and A.Br.Ames subgroups in the CBaGID

Taking together the *B. anthracis* isolate from the index patient and 18 DNA templates extracted from vesicular fluid specimens in the Shaanxi anthrax outbreak, a total of 19 specimens tested positive using RT-PCR assays. These DNA templates were subtyped by canSNP, MLVA15, and SNR methods. However, only nine DNA templates get all the results in the three methods. The specimens from these nine patients were clustered together and identified as the A.Br.001/002 canSNP subgroup and MLVA15-31 genotype (i.e. the 31 genotype in MLVA scheme with 15 VNTR loci). Figure 2 shows the genetic relationship between outbreak-related DNA samples and other *B.anthracis* isolates from China or other countries using the canSNP and MLVA methods. Figure 2 also shows the dendrogram of the other representative isolates identified as A.Br.001/002 or A.Br.Ames subgroups (closely related A.Br.001/002 subgroups) in China.

A.Br.001/002 is the dominant subgroup in China (106/190) and is scattered throughout mainland China, including in the Xinjiang, Guangxi, Inner Mongolia, Shaanxi, Qinghai, Hebei, Henan, Shandong, Yunnan, Liaoning, Shanghai, Beijing, Jiangxi, Guizhou, Guangdong, Jiangsu, and Xizang (Tibet) provinces (see Additional file 3: Table S1). Comparing the 15 loci profiles of MLVA15 scheme in all isolates in the CBaGID, the recent outbreak-related isolates were grouped together with nine isolates isolated from four different provinces in different years(Their genotype of MLVA15 was MLVA15-31), i.e. three isolates from the Inner Mongolia Autonomous Region (one strain isolated in 2012, two in 1998); two isolates from Guangxi (1994); two isolates from Liaoning (2011); and two isolates from Xinjiang Autonomous Region (one in 1995, one in 1981). In addition, eight isolates described in a previous study [4] were clustered into MLVA15-31 genotype (Fig. 1 and Additional file 3: Table S1), i.e. three isolated from Shandong (1963–1964), two from Inner Mongolia (1962), one from Gansu (1978), and two isolated in 1957 (exact area(s) unknown) (Additional file 3: Table S1). Only one strain (SN38, isolated in 1953 from a goat) from the Shaanxi province existed in the database. However, the clustering results showed that there were two loci different among the SN38 and the outbreak-related isolates in Shaanxi in 2015 (see Additional file 3: Table S1).

### Source tracking using the SNR analysis

**Fig. 3:**
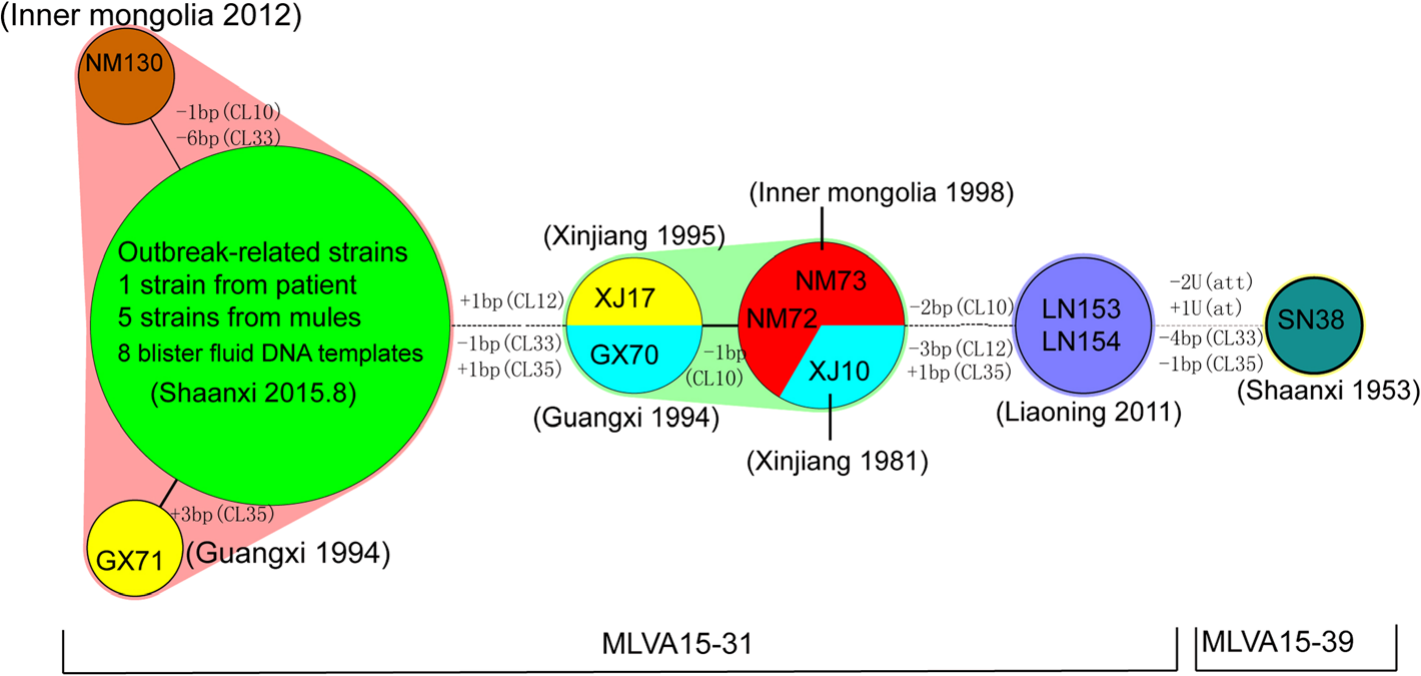
Minimum spanning tree of *B. anthracis* strain in MLVA15-31. Each circle denotes a SNR SGT and the halos surrounding the various subgenotype denote the complexes created by the maximum neighbor distance with two changes

The SNR analysis is shown in Fig. 3. It identified six subgenotypes (SGTs) among the isolates in the MLVA15-31 genotype, which suggests that isolates with the same MLVA genotype could be differentiated from each other by SNR markers. The nine vesicular fluid specimens shared the same SNR profiles (SGT-I) with the index patient’s strain (WCS1). Meanwhile, five strains isolated from diseased mules were clustered together with the index patient’s strain (SGT-I). The results showed that the outbreak-related strains probably originated from a single source. However, the strain SN38 isolated from a goat in Shaanxi in 1953 showed different SNR profiles (two SNR loci). Such observation suggests that there is no genetic connection between the two *B. anthracis* outbreaks, even though they both occurred in Shaanxi. Among the isolates clustered in the MLVA15-31 genotype, two isolates had closer relationship with the outbreak-related strains, i.e., GX71 isolated from Guangxi province and classified as SGT-II had only one locus different from the outbreak-related strains in CL35 (adding three A/T base pairs). However, this strain was isolated in 1994 in a location that is not adjacent to Shaanxi. Another strain NM130 (SGT-III) isolated from Inner Mongolia, an area adjacent to Shaanxi, had two loci different (CL10 and CL33) with the outbreak-related strains (Fig. 3 and Additional file 4: Table S2).

## Discussion

Human anthrax is an old and persistent problem in many rural regions of China. 200–500 anthrax cases have been reported each year in recent decade, according to national anthrax surveillance data (The surveillance and reporting data for notifiable infectious disease, China CDC). Anthrax is categorized as a Class B infectious disease under China’s Law on the Prevention and Treatment of Infectious Diseases. However, outbreaks related to pulmonary anthrax have been administrated according to the regulations of Class A infectious diseases in China (two diseases that belong to Class A are plague and cholera) because this type of anthrax could be transmitted by inhaling aerosol and delayed treatment always leads to death. Poor coverage among livestock of the *B. anthracis* vaccination and a lack of supervision of diseased and dead animals contributes to animal anthrax outbreaks in China. Human anthrax outbreaks have always been associated with the butchering of anthrax-infected animals and exposure to infected meat and animal byproducts. In many rural regions of China, sick and dying animals are often slaughtered, and the meat of dead animals is usually consumed by villagers or even sold at markets to eliminate economic losses. Five dead mules were sold during this outbreak and only three animal carcasses were retrieved by the local government, while the others were not yet retrieved by the end of our investigation.

A strengthened national anthrax surveillance system (including 11 provinces) in mainland China has been in place since 2005. However, the Shaanxi province is not included. According to national epidemiological surveillance data of Shaanxi and Yan’an prefecture, Shaanxi has had a lot of human anthrax cases prior to the 1990s, especially in middle areas such as Weinan and Xianyang prefectures, etc. However in the 21^st^ century, only sporadic cases have been reported. Yan’an was not a high-risk area for anthrax in Shaanxi; 60 anthrax cases were reported between 1970 and 1981, and no case was reported after 1982. Only one human clinical anthrax case was reported in 1973 in Ganquan county. However, the outbreak reported in this paper resulted in 19 infected patients and killed at least 15 animals, which suggests that more provinces and areas should be included in the national anthrax surveillance system, even though these areas might have historically had comparatively low anthrax occurrence rates.

Anthrax is generally diagnosed by bacterial culture, as well as four-fold serum antibody titer increase or positive conversion. However, culture often fails due to antibiotic use. DNA templates extracted from clinical specimens could be used for RT-PCR analysis, which could provide laboratory evidence to identify anthrax in a timely manner. These templates could also be directly used in molecular typing or source tracking based on PCR amplification. They could also greatly facilitate the comparison of genetic characteristics in investigations of anthrax outbreaks.

The precision of subtyping assays is important for epidemiologic and forensic investigations [3]. Different molecular subtyping methods have different distinguishing ability and could be used for different purposes. *B. anthracis* is a relatively genetically homogeneous bacteria species [4]. A previous canSNP study indicated that five different sublineages/subgroups in China are included in 12 worldwide canSNP genotypes [4, 10]. The *B. anthracis* strain isolated from Shaanxi in 2015 was found to belong to the A.Br.001/002 subgroup. The A.Br.001/002 subgroup is the dominant subgroup in China [4] and another study suggests that it may have even originated in China [10]. Comparing with all the strains in the CBaGID, 17 isolates isolated from at least six provinces shared the same canSNP and MLVA15 genotype (MLVA15-31) with the outbreak-related strains. Ten isolates from our collection could be further identified as six SGTs. This observation indicates that even though the MLVA showed a comparatively high resolution, it did not meet the needs for detailed source tracking of an outbreak-related infection. The SNR method with more polymorphism should be used for source tracking. Such methods could differentiate very closely related strains and could be useful molecular epidemiological tools in outbreak or bioterrorism events [15].

In this study, no isolates had the same SNR profiles as the outbreak-related strains and only two isolates had a comparatively closer relationship with the outbreak-related strains. We speculate that the anthrax outbreak in Shaanxi province in 2015 occurred locally because the diseased animals and subsequent human cases were all located in the same valley, no animal or human anthrax was reported in a nearby valley; No livestock were imported into the suffered valley by trading shortly before the outbreak; and no genetically similar stains were found in an adjacent province in 2015. *B. anthracis* spores may spread to a new geographic region through water, insects, wild animals, birds, and contamination from body fluids of infected animals [16], and spores could persist for long periods of time in the environment. We want to emphasize that *B. anthracis* in the CBaGID were limited and that it was difficult to isolate the *B. anthracis* strain as the pathogen loads were lower after the administration of antibiotics. This means that we could not obtain all subtyping results from these anthrax cases. Therefore, we cannot exclude the possibility that *B. anthracis* strains might have been imported into the outbreak area from other areas in the same timeframe. In addition, with the development of next generation sequencing, genome-based analysis becomes a powerful tool for the investigation of outbreaks of infectious disease, such work could be practiced in future.

## Conclusions

In summary, an anthrax outbreak occurred in Shaanxi province in 2015 were described in this study and three molecular subtying methods were used to investigate the possible source and genetic source tracking. Such analysis is helpful for us to judge the relationship of anthrax cases and could help us to determine whether the disease agents are biological attacks, emerging agents or more familiar pathogens in future.

## Additional files

Additional file 1: Multilingual abstracts in the five official working languages of the United Nations. (PDF 690 kb)
Additional file 2: Table S3. All cases involved in the investigation and their laboratory results. (XLS 21 kb)
Additional file 3: Table S1. The genotypes and profiles of MLVA15. The nomenclature of genotypes of MLVA15, as according to Keim Genetics Lab ID Designation. For the new genotypes, the nomenclatures labeled “CN” were used. (XLS 45 kb)
Additional file 4: Table S2. The SGTs of SNR (CL10, CL12, CL33, CL35) in MLVA15-31 strains. (XLS 28 kb)

## Abbreviations

canSNP: canonical single nucleotide polymorphisms
CBaGID: Chinese *B. anthracis* Genetic Information Database
CDC: Center for disease control and prevention
CE: capillary electrophoresis
MLVA: multiple-locus variable-number tandem repeat analysis
PA: anti-protective antigen
RT-PCR: real-time polymerase chain reaction
SGT: subgenotype
SNR: single nucleotide repeat
VNTR: variable-number tandem repeat
WHO: World health organization
