# Supplementary material for: Genetic source tracking of an anthrax outbreak in Shaanxi province, China
Source: Infect Dis Poverty. 2017 Jan 17;6:14. doi: 10.1186/s40249-016-0218-6 (PMC5240257; doi:10.1186/s40249-016-0218-6)
Supplement: Additional file 1: — Multilingual abstracts in the five official working languages of the United Nations. (PDF 690 kb) [file 40249_2016_218_MOESM1_ESM.pdf]

Translation of the abstract into the five official working languages of the United Nations

تتبع المصدر الوراثي لتفشي الجمرة الخبيثةفي مقاطعة شنشي، بالصين

دونغ لى ليو، وجيان تشون وى، وتشيو لان تشن، وشيويه يونيو جو، وإن مين تشانغ، ولى هي، وشو دونغ ليانغ،وجو زو ما، وتي تساو تشو، وون وو بين، ووى ليو، وكاي ليو، ويي شي، وجيان يونيو جي، وهوى خوان تشانغ، ولين ما، وفا شين تشانغ، وتشى كاي تشانغ، وهانغ تشو، وهونغ جي يو، وبياو كان, وجيان جو زو، وفنغ ليو ويى لي

تلخيص

معلومات عامة: الجمرة الخبيثة هي مرض معد حيواني المنشأ حاد تسببه بكتيريا تعرف باسم *عصيات الجمرة الخبيثة*. وقد جرى الإبلاغ منذ 26 يوليو - 8 أغسطس عام 2015، عن اشتباه تفشي 20 حالة من الجمرة الخبيثة الجلدية في مقاطعة جانكان بمقاطعة شنشي في الصين. وقد أجري تحليل تتبع المصدر الوراثي لتفشى مرض الجمرة الخبيثة عن طريق الوسائل الوبائية الجزيئية في هذه الدراسة.

الطرق: استخدم ثلاثة ثلاث طرق الكتابة الجزيئية، وهي تعدد الأشكال الأحادي القانوني للنوكليوتيدات (canSNP) والتحليل المتكرر للعدد متغير ((MLVA، والتحليل الأحادي لتكرار النوكليوتيدات (SNR) من أجل البحث في مصدر محتمل للانتقال وتحديد العلاقة الوراثية بين السلالات المعزولة من الحالات البشرية والحيوانات المريضة أثناء تفشي المرض.

النتائج: تم تجميع خمس سلالات معزولة من البغال المريضة مع عازلات المرضى باستخدام كتابة scanSNAP وMLVA. تنتمي أنساب الجمرة الخبيثة المتفشية المسببة لمرض *الجمرة الخبيثة ب* إلى المجموعة الفرعية A.Br.001/002 canSNP والنمط الجيني 31) MLVA15-31 نمط جيني في مخطط MLB 15). وبسبب تجميع تسع عازلات من أربع محافظات أخرى في الصين إلى جانب سلالات ذات صلة بتفشي المرض بطريقة canSNP (A.Br.001 / 002 فرعية) وطريقة MLVA15 (والنوع الجيني (MLVA15-31، ومازال التحليل الآخر باستخدام SNR (CL10، CL12، CL33 مستخدمًا وCL35) لتتبع مصدر تفشي المرض، وتشير النتائج بأنه من المرجح إصابة هؤلاء المرضى بالجمرة الخبيثة ربما كانت مصابة بنفس استنساخ الكائن الممرض.

الاستنتاجات: جرى استخلاص أن تفشى مرض الجمرة الخبيثة حدث في مقاطعة شنشى، الصين في عام 2015 كان مجرد حدث محلي.

Translated from English version into Arabic by Fathia Sobhi, through

TRANSLATORS
WITHOUT BORDERS

Translation of the abstract into the five official working languages of the United Nations

陕西炭疽爆发疫情的遗传溯源分析

刘东立，魏建春，陈秋兰，郭学军，张恩民，贺莉，梁旭东，马国柱，周体操，殷文武，刘炜，刘凯，石一，纪建军，张慧娟，马琳，张发信, 张志凯，周航，余宏杰，阚飙，徐建国，刘峰，李伟

摘要
引言：炭疽是一个急性人兽共患传染病。2015 年 7 月 26 至 8 月 8 日，陕西省甘泉县暴发一起皮肤炭疽暴发疫情，报告病例 20 例。本研究利用分子流行病学方法开展病原学溯源调查。
方法：应用三种炭疽芽孢杆菌分子分型方法：即标准单核苷酸多态性分析（canonical single nucleotide polymorphisms, canSNP）、多位点串联重复序列分析（multiple-locus variable-number tandem repeat analysis, MLVA）、单碱基重复分析（single nucleotide repeat，SNR）调查此次疫情的感染来源，同时开展暴发相关菌株的遗传溯源分析。
结果：陕西 2015 年炭疽暴发疫情中分离的 1 株病人和 5 株病死骡的炭疽芽孢杆菌均被鉴定为 A.Br.001/002 群（canSNP）和 MLVA15-31 基因型。比对分子分型数据发现，来源于中国四个省份的 9 株炭疽芽孢杆菌和陕西炭疽暴发菌株同属 MLVA15-31 基因型。因此利用更具多态性的 SNR 方法(指标包括 CL10, CL12, CL33, and CL35)进一步开展暴发相关菌株的遗传溯源分析，结果提示此次炭疽暴发中人和动物炭疽为一次相同感染来源的爆发疫情。
结论：2015 年陕西炭疽疫情为非输入性本地暴发疫情。

Translated from English version into Chinese by Wei Li

Recherche génétique de la source d'une épidémie d'anthrax dans la province de Shaanxi en Chine

Dong-Li Liu, Jian-Chun Wei, Qiu-Lan Chen, Xue-Jun Guo, En-Min Zhang, Li He, Xu-Dong Liang, Guo-Xhu Ma,Ti-Cao Zhou, Wen-Wu Yin, Wei Liu, Kai Liu, Yi Shi, Jian-Jun Ji, Hui-Juan Zhang, Lin Ma, Fa-Xin Zhang, Zhi-Kai Zhang, Hang Zhou, Hong-Jie Yu, Biao Kan, Jian-Guo Xu , Feng Liu, Wei Li

Résumé
Contexte : L'anthrax est une zoonose aigüe causée par la bactérie *Bacillus anthracis.* Entre le 26 juillet et le 8 août 2015, une épidémie a causé 20 cas suspectés d'anthrax cutané en Chine, dans la province du Shaanxi, circonscription de Ganquan. La recherche génétique de la source de cette épidémie a été réalisée par des méthodes d'épidémiologie moléculaire dans le cadre de la présente étude.
Méthodes : Trois méthodes d'identification moléculaire (polymorphisme de nucléotides individuels [canSNP], analyse de locus VNTR multiples [MLVA] et répétition de nucléotides individuels [SNR]) ont été utilisées pour remonter à la source possible de transmission et identifier les liens génétiques entre les souches isolées sur les cas humains et animaux pendant l'épidémie.
Résultats : Cinq souches isolées sur des mules malades ont été regroupées avec des isolats de patients en vue de l'identification par canSNP et MLVA. Les lignées de *B. anthracis* à l'origine de

l'épidémie en question appartenaient au sous-groupe canSNP A.Br.001/002 et au génotype MLVA15-31 (le 31ème génotype dans le schéma de génotypage MLVA15). Comme neuf isolats provenant de quatre autres provinces de Chine ont été regroupés avec des souches impliquées dans l'épidémie pour les analyses par canSNP (sous-groupe A.Br.001/002) et MLVA15 (génotype MLVA15-31), une autre analyse par SNR (CL10, CL12, CL33, and CL35) a été utilisée pour remonter à la source de l'épidémie. Les résultats suggèrent que les patients infectés au cours de l'épidémie l'ont probablement été par le même clone de la bactérie pathogène.
Conclusions : Nous en déduisons que l'épidémie d'anthrax qui s'est produite dans la province chinoise du Shaanxi en 2015 était un évènement local.

Translated from English version into French by Suzanne Assenat, through

Генетический анализ эпидемии сибирской язвы в провинции Шаанкси, Китай

Донг-Ли Лью, Джиан-Чун Вэй, Куи-Лан Чен, Ксуе-Джун Гуо, Ен-Мин Дзанг, Ли Хе, Ксу-Донг Льянг, Гуо-Ксху Ма,Ти-Као Жоу, Вэн-Ву Йин, Вэй Лью, Кай Лью, Йи Ши, Дзьян-Джун Джи, Хуи-Джуан Жанг, Лин Ма, Фа-Ксин Жанг, Жи-Кай Жанг, Ксанг Жоу, Хонг-Джие Ю, Бьяо Кан, Джиан-Гуо Ксу , Фенг Лью, Вэй Ли

Резюме

Краткая информация: Сибирская язва – острое природно-очаговое инфекционное заболевание, вызываемое бактериями *Bacillus anthracis.* Подозрения на 20 случаев заболевания кожной формой сибирской язвы зафиксированы в районе Ганкван, провинции Шаанкси в Китае с 26 июля по 8 августа 2015. В рамках данного исследования проведен генетический анализ с применением молекулярных эпидемиологических методов.
Методы: Три разных метода молекулярного типирования, а именно: классический метод определения однонкулеотидного полиморфизма (canSNP), метод мультилокусного VNTR-анализа (MLVA), и метод определения повтора единичного нуклеотида (SNR) использовались для определения возможных путей заражения, а также для определения генетической взаимосвязи между штаммами, выделенными у человека и умерших животных, во время вспышки заболевания.
Результаты: Пять штаммов, изолированных от зараженных мулов, были сгруппированы с изолированными от человека штаммами с помощью методов canSNP и MLVA. Клеточные линии возбудителя *B. anthracis* принадлежали к подгруппе A.Br.001/002 (canSNP) и генотипу MLVA15-31 (генотип 31 в схеме MLVA15). Несмотря на то, что 9 изолятов сравнивались с полученными в других 4х провинциях Китая при помощи canSNP (A.Br.001/002 подгруппа) и MLVA15 (MLVA15-31 генотип), дополнительно был проведен анализ SNR (CL10, CL12, CL33, и CL35) для изучения особенностей эпидемии, и результаты указывали на то, что пациенты, заболевшие во время упомянутой вспышки сибирской язвы, были инфицированы

тем же клоном микроорганизмов патогенного вида.
Выводы: Было определено, что вспышка сибирской язвы в провинции Шаанкси в 2015 году в Китае носила местный характер.

Translated from English version into Russian by Ms Zhdanova, through

Rastreo del origen genético de un brote de carbunco en la provincia de Shaanxi (China)

Dong-Li Liu, Jian-Chun Wei, Qiu-Lan Chen, Xue-Jun Guo, En-Min Zhang, Li He, Xu-Dong Liang, Guo-Xhu Ma,Ti-Cao Zhou, Wen-Wu Yin, Wei Liu, Kai Liu, Yi Shi, Jian-Jun Ji, Hui-Juan Zhang, Lin Ma, Fa-Xin Zhang, Zhi-Kai Zhang, Hang Zhou, Hong-Jie Yu, Biao Kan, Jian-Guo Xu , Feng Liu, Wei Li

Resumen

Antecedentes: El carbunco es una enfermedad infecciosa zoonótica aguda producida por el *Bacillus anthracis.* Del 26 de julio al 8 de Agosto de 2015, en el condado de Ganquan, en la provincia de Shaanxi (China), se dieron 20 presuntos casos de carbunco cutáneo. En este estudio, el rastreo del origen genético del brote de carbunco se realizó mediante métodos epidemiológicos moleculares.
Métodos: Para investigar la posible fuente de transmisión e identificar la relación genética entre las cepas aisladas de personas y de animales durante el brote, se utilizaron tres métodos de tipado molecular, concretamente el análisis de polimorfismos normales de un solo nucleótido (canSNP, por sus siglas en inglés), el análisis del número variable de repeticiones en tándem en varios locus (MLVA, por sus siglas en inglés) y el análisis de las repeticiones de un solo nucleótido (SNR, por sus siglas en inglés).
Resultados: Se aislaron cinco cepas de mulas enfermas y se agruparon con las cepas de los pacientes mediante tipado de canSNP y MLVA. La estirpe de *B. anthracis* causante de este brote pertenecía al subgrupo A.Br.001/002 de los canSNP y al genotipo MLVA15-31 (el genotipo 31 en el esquema de MLVA15). Debido a que con el análisis de canSNP (subgrupo A.Br.001/002) y el MLVA15 (genotipo MLVA15-31), nueve cepas aisladas de otras cuatro provincias chinas se agruparon con las cepas procedentes del brote, se realizó otro análisis de las SNR (CL10, CL12, CL33 y CL35) para identificar el origen del brote, y los resultados indicaron que estos pacientes del brote de carbunco probablemente se infectaron con el mismo clon patógeno.
Conclusiones: Se concluyó que el brote de carbunco que tuvo lugar en la provincial de Shaanxi (China) en 2015 fue un caso aislado.

Translated from English version into Spanish by Mar Jiménez Quesada, through
